# Radiological features of basal ganglia germinoma: a case report and early-stage alerts

**DOI:** 10.3389/fonc.2024.1387813

**Published:** 2024-12-16

**Authors:** Quyen Thi To Nguyen, Phuc Van Nguyen, Phuong Hoang Ho, Khiem Tan Le, Thanh Anh Le, Truc Thi Thuy Nguyen

**Affiliations:** Diagnostic Imaging Center, Tam Anh General Hospital, Ho Chi Minh City, Vietnam

**Keywords:** germinomas, basal ganglia, early stage, case report, magnetic resonance imaging

## Abstract

Basal ganglia germinomas are uncommon neoplasms. Basal ganglia germinomas exhibit high sensitivity to both radiation therapy and chemotherapy. In contrast, surgery is the standard treatment for most primary brain tumors (such as gliomas, which are the most common tumors in the pediatric basal ganglia region). A 21-year-old male patient was admitted to our hospital because of unexplained right-sided hemiparesis for two years. Biomarkers indicating germ cell tumors are typically negative. On the initial MRI, the abnormalities were nonspecific, causing no suspicion of a tumor. Therefore, the patient did not receive an earlier diagnosis or treatment. His brain MRI revealed a mass with more apparent features one year later. Based on the imaging characteristics of conventional and advanced MR images, the preoperative radiological diagnosis was highly likely to reveal germinomas. The patient then underwent a biopsy and received appropriate treatment. Despite treatment, his symptoms only partially improved. Accurate preoperative diagnosis is crucial for ensuring that patients receive appropriate treatment and to help avoid more invasive surgery. Additionally, early identification of germinomas is also important for improving long-term patient outcomes and preventing tumor spread due to delayed diagnosis. Therefore, we aim to review and report this case to assist radiologists in recognizing and becoming familiar with the early imaging signs of basal ganglia germinoma.

## Introduction

Intracranial germ cell tumors (GCT) are uncommon neoplasms (accounting for 1-2% of all primary brain tumors), of which germinomas are the most common histological type. Intracranial germinomas have a distinct predilection for midline structures (1, 2). They are mostly found in the pineal region, followed by the suprasellar region. In rare cases, it may occur in the basal ganglia region, accounting for 5-10% of all intracranial germinomas (2, 3). It usually occurs in male adolescents in the second decade of life, with a higher incidence in Asian populations (4).

The clinical manifestations of germinoma vary depending on the tumor’s location. The basal ganglia, a critical region of the brain, plays a key role in motor control, cognitive functions, and emotional regulation. Consequently, a tumor in this area may result in progressive neurological deficits, including hemiparesis, movement disorders, and impairments in cognition, memory, language, and behavior (3, 5–7).

Basal ganglia germinomas exhibit high sensitivity to both radiation therapy and chemotherapy. Therefore, in the treatment of germinoma, radiation therapy is recommended following a histological diagnosis (8–10). In contrast, surgery is the standard treatment for most primary brain tumors (such as gliomas, which are the most common tumors in the pediatric basal ganglia region) (11). Accurate preoperative diagnosis is crucial for ensuring that patients receive appropriate treatment and to help avoid more invasive surgery. Additionally, early identification of germinomas is also important for improving long-term patient outcomes and preventing tumor spread due to delayed diagnosis. The early diagnosis of basal ganglia germinoma is particularly challenging due to its rarity, the overlap of clinical symptoms with common neurological disorders, and the presence of subtle or atypical imaging features, leading to delays in diagnosis. Therefore, recognizing the early imaging features of basal ganglia germinoma is essential for radiologists, enabling a high index of suspicion and often necessitating advanced imaging techniques or biopsy for a definitive diagnosis.

In this case report, we describe the clinical presentation, laboratory results, and radiological findings of a young adult patient who was presented to our radiology center and had subsequent histological evidence of germinoma in the basal ganglia.

## Case description

**Figure 1:**
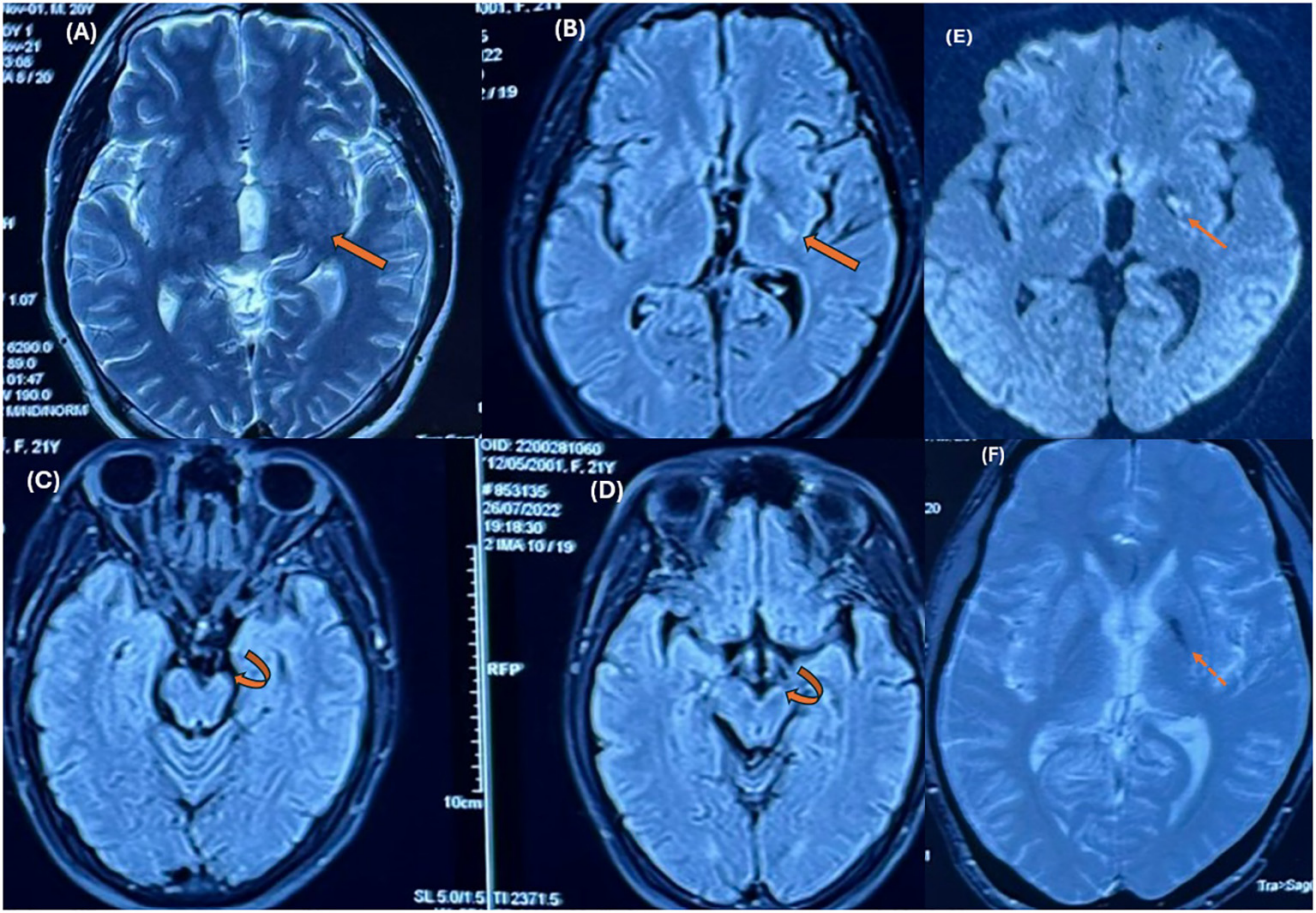
MR images were obtained at other institutions one year prior. Axial T2-weighted **(A)** and FLAIR images **(B)** show an ill-defined hyperintense lesion in the posterolateral region of the left basal ganglia (thick arrows). Ipsilateral hemiatrophy can be seen in the cerebral peduncle (curved arrows in **C, D**) and pons compared to the opposite normal-appearing side. Axial T2* GRE images **(F)** demonstrate hypointensity in the left basal ganglia. Focal hyperintensity is also noted on DWI **(E)**. * T2W, T2 – Weighted, FLAIR, Fluid-Attenuated Inversion Recovery; GRE, Gradient echo sequences; DWI, Diffusion-weighted imaging.

A 21-year-old male patient was admitted to our hospital because of unexplained right-sided hemiparesis for two years. In 2022, one year prior to hospitalization, he underwent brain magnetic resonance imaging (MRI) studies at another hospital. The initial MR findings were a small hypointense area in the left basal ganglia on the T2* gradient echo (GRE) sequences without mass effect and the loss of volume of the ipsilateral cerebral peduncle and pons compared to the opposite normal-appearing side (**Figure 1**). No other abnormalities were noted. At that time, the radiologist suspected an acute demyelinating lesion. However, due to its nonspecific nature, no additional studies were performed, and no treatment was administered.

Currently, he has presented to our hospital with gradually worsening right-sided hemiparesis compared to a year ago. Neurological examination reveals right-sided hemiparesis with upper limb muscle strength at grade 4/5 and lower limb at grade 3/5, without sensory or language disturbances. No other abnormalities were noted on clinical examination. The patient has no significant medical history or family history.

The laboratory results demonstrated that serum α-fetoprotein (AFP) was 0.75 IU/mL, β-human chorionic gonadotropin (β-hCG) was 0.2 mIU/mL, and lactate dehydrogenase (LDH) was 157.3 U/L, all within the normal reference ranges.

**Figure 2:**
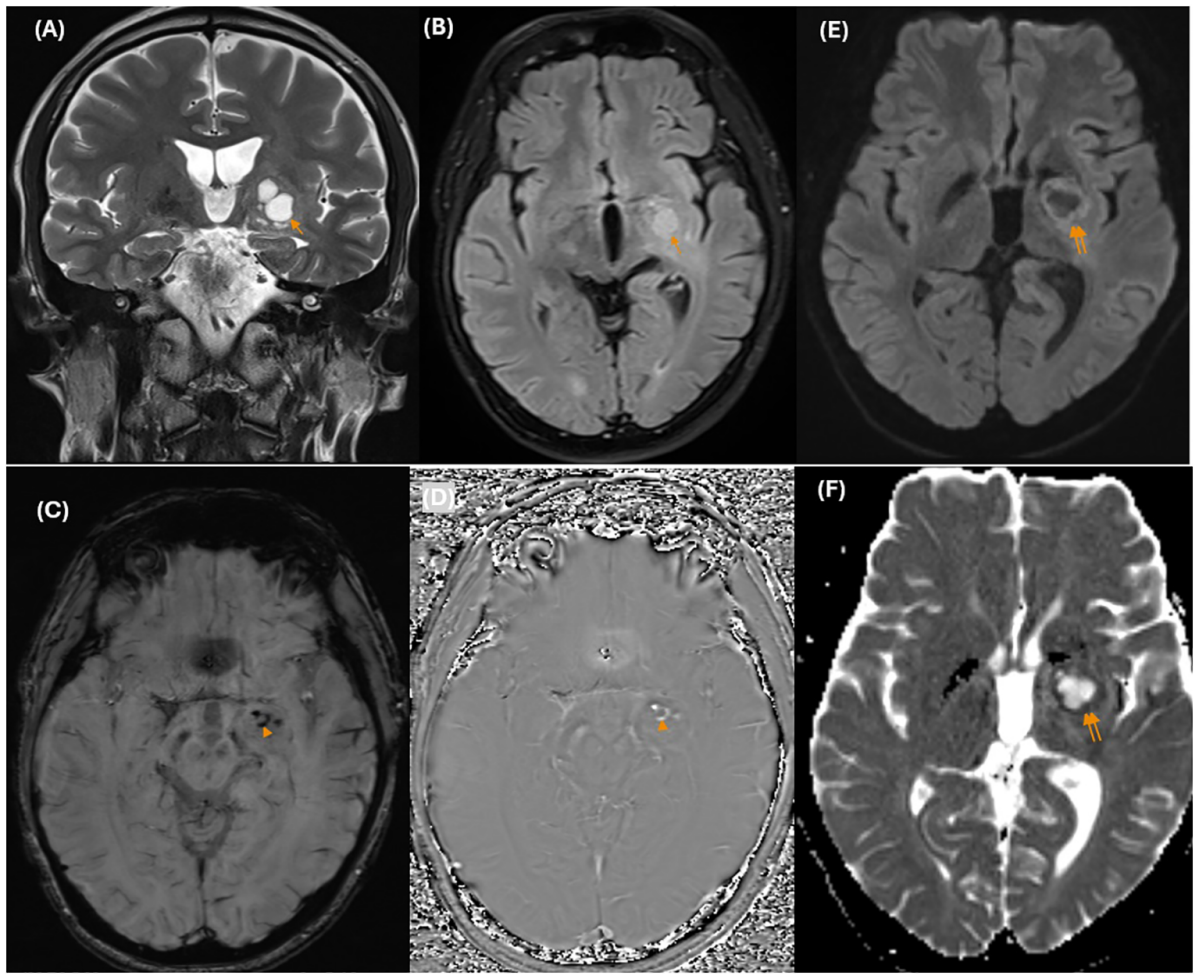
On conventional MR images, coronal T2-weighted images **(A)** show an ill-defined hyperintense mass centered on the left basal ganglia with cystic changes (arrow) that are not completely suppressed on FLAIR images **(B)**. There are several hypointense dots (arrowhead) on the SWI images **(C)**, and the corresponding hyperintensity on the phase image **(D)** represents hemorrhage. There was hyperintensity on DWI **(E)** and corresponding hypointensity on the ADC maps **(F)**. *MR, magnetic resonance; T2W, T2 – Weighted; FLAIR, Fluid-Attenuated Inversion Recovery; SWI, Susceptibility weighted imaging; DWI, Diffusion-weighted imaging; ADC, Apparent diffusion coefficient.

**Figure 3:**
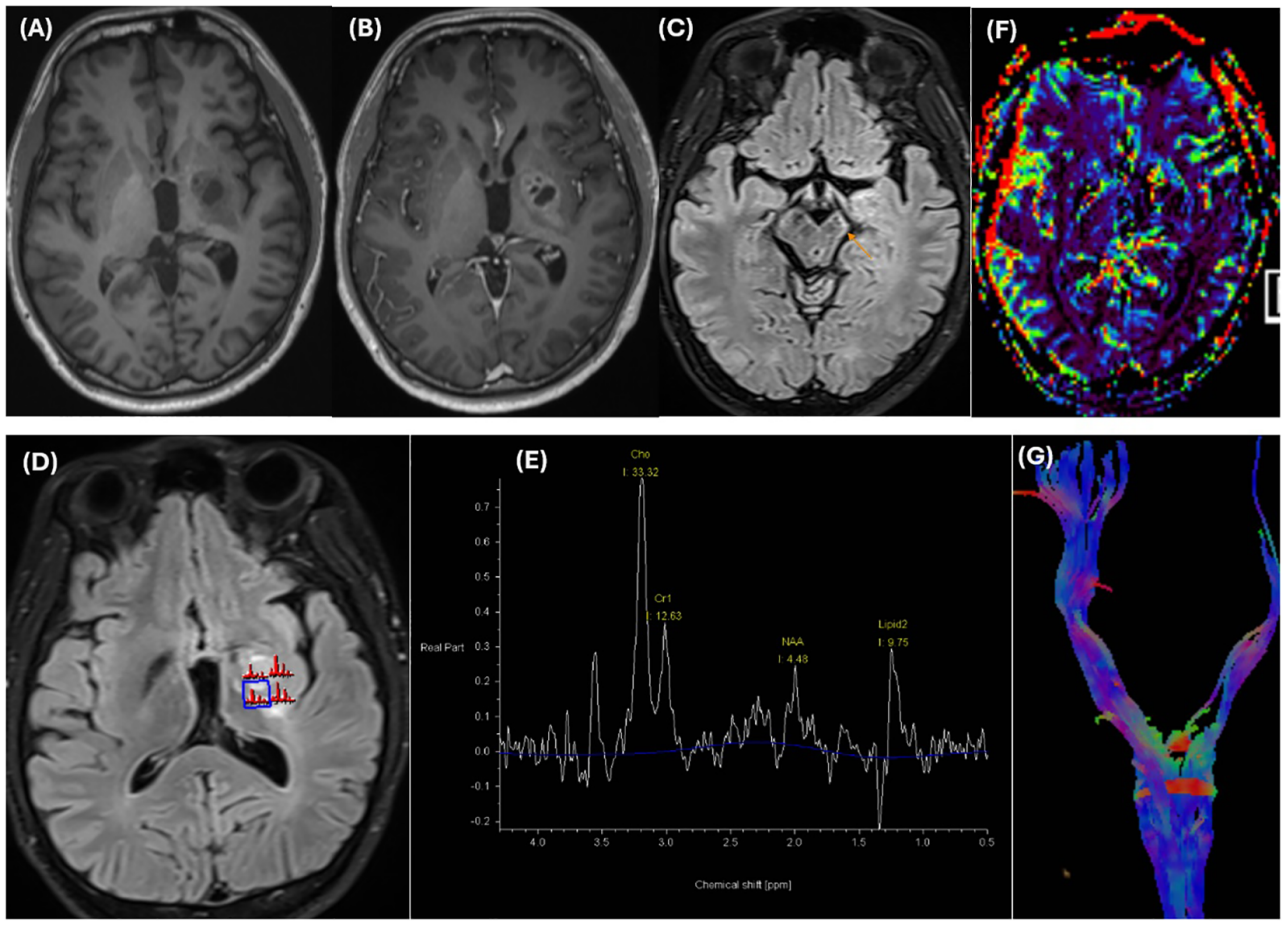
The mass is isointense on T1-weighted images **(A)** and strongly but heterogeneously enhances on T1 MPRAGE postcontrast images **(B)**. Significant volume loss of the cerebral peduncles (thin arrow) and pons in the left hemisphere, likely representing Wallerian degeneration, is noted in axial FLAIR images **(C)**. MRS images **(D, E)** reveal an elevated Choline peak and a decreased NAA peak in the enhancing components. A prominent lipid peak was also noted. Dynamic susceptibility contrast perfusion-weighted imaging (DSC-PWI) **(F)** reveals a lower rCBV than did the contralateral normal-appearing white matter. Tractography **(G)** reveals volume loss of the left corticospinal tract and decreased FA in the posterior limb of the internal capsule on the color-encoded FA map (not shown). T1W, T1 – Weighted; FLAIR, Fluid-Attenuated Inversion Recovery; MRS, Magnetic resonance spectroscopy; NAA, N-acetylaspartate; DSC-PWI, Dynamic susceptibility contrast perfusion-weighted imaging; rCBV, Relative cerebral blood volume; FA, Fractional anisotropy.

He underwent MRI once again. Brain MRI revealed an ill-defined mass centered on the basal ganglia involving the putamen, globus pallidus, caudate nucleus, lateral thalamus, posterior limb of the internal capsule, medial temporal lobe, and cerebral peduncle in the left hemisphere. There was mild edema surrounding the mass with minimal mass effect. The mass was isointense on T1-weighted (T1W) images and hyperintense on T2-weighted (T2W) images, with cystic changes that were not completely suppressed on fluid-attenuated inversion recovery (FLAIR) images. There were several hypointense dots on the susceptibility weighted imaging (SWI) sequence and corresponding hyperintensity on the phase images representing hemorrhage. There was restricted diffusion of the solid portions with low ADC values (ADC_mean_ value approximately 0.62 ×10^-3^ mm^2^/s) (**Figure 2**). The mass showed moderate to strong but heterogeneous enhancement after contrast agent administration. A significant decrease in the volume of the cerebral peduncles and pons in the left hemisphere, likely representing Wallerian degeneration, was also noted (**Figure 3**).

In addition, on multivoxel magnetic resonance spectroscopy (MRS), there was an elevated choline (Cho) peak and a decreased N-acetyl aspartate (NAA) peak (with an NAA/Cr ratio and Cho/Cr ratio of 0.34 and 2.43, respectively) in the enhancing components. In addition, a prominent lipid peak potentially related to necrosis and cystic degeneration was also noted. Dynamic susceptibility contrast perfusion-weighted imaging (DSC-PWI) revealed a lower relative cerebral blood volume (rCBV) than did the contralateral normal-appearing white matter. The tractography images showed a volume loss of the left corticospinal tracts and a decreased fractional anisotropy (FA) value (mean value is approximately 260 mm^2^/sec) in the posterior limb of the internal capsule (**Figure 3**). This finding revealed Wallerian degeneration and reflected the infiltrative and destructive growth pattern of the tumor. Given the patient’s age, clinical presentation, laboratory findings, and imaging characteristics, a diagnosis of a germ cell tumor in the basal ganglia is strongly suspected.

This was a biopsy-proven germinoma.

## Discussion

Germinomas are tumors of young patients, with approximately 90% of patients being younger than 20 years old at the time of diagnosis (8). Germinomas that occur in the basal ganglia are uncommon entities (3). Germinomas in the basal ganglia typically present with slow progressive neurological deficits, including hemiparesis, movement disorders, and impairments in cognition, memory, language, and behavior (3, 5, 7, 12). Symptoms can be nonspecific and overlap with other neurological conditions, leading to delays in diagnosis.

In contrast to non-germinomatous germ cell tumors (NGGCT), which frequently exhibit positive biological markers, most germinomas are nonsecretory. Consequently, biomarkers commonly used to indicate germ cell tumors, such as serum or cerebrospinal fluid AFP, β-hCG, and LDH, are typically negative (8). In certain cases of germinoma containing syncytiotrophoblast components, β-hCG is secreted, resulting in elevated β-hCG levels (1, 6, 8). It has been reported that elevated β-hCG levels in germinomas are associated with an increased risk of recurrence (6). Due to the complexity of tumor components, AFP and β-hCG levels in the NGGCT showed significant variability. Notably, some NGGCT were also non-secretory, exhibiting normal tumor marker levels. This variability can complicate the accurate diagnosis of GCT subtypes (1, 8).

The diagnosis of basal ganglia germinoma at an early stage is usually difficult, owing to its rarity and nonspecific clinical presentation. Imaging is usually the first clue to the diagnosis, although the findings are subtle and may be overlooked or misinterpreted as other entities. Early MRI findings may exhibit small areas of signal changes in the basal ganglia without mass effects (3, 13). The lesions show variable signal intensities in T1W images, hyperintensities in T2W/FLAIR images, and minimal or no enhancement. Mild to moderate diffusion restriction is present (3, 12–14). Abnormal signal changes in the basal ganglia, as observed in this case, require differential diagnosis from non-tumorous lesions such as ischemia/infarction, demyelination, encephalitis, focal areas of signal intensity (FASI), or unidentified bright objects (UBO) in Neurofibromatosis type 1 (NF1), ect. However, cerebral infarction typically presents with a more acute clinical progression, and the absence of vascular stenosis or occlusion on angiographic imaging can assist in distinguishing these conditions (15). Furthermore, the T2* sequences (SWI or T2* GRE) show a hypointense region in the basal ganglia. This can help in differentiating between germinomas and nontumorous conditions such as infarction and demyelination, ect. in which the signal drop on the SWI sequence is usually not remarkable (13). Some reports show that hypointensity in the basal ganglia on SWI sequences (reflecting hemorrhage or iron deposition in the tumor) is very important and helpful in identifying the early stages of germinomas (1, 4, 13, 16).

Additionally, hemiatrophy in the ipsilateral hemisphere and brainstem, which may represent Wallerian degeneration, is thought to characterize germinomas arising from the basal ganglia or thalamus (1, 8). This is the result of the involvement of the internal capsule and thalamic ganglion cells due to any pathology. Germinomas typically involve the internal capsule and periphery of the thalamus despite the very small size of the tumors. Additionally, their slow and infiltrative growth leads to this type of atrophy of the brainstem, which is rarely observed in other fast-growing tumors or non-tumorous conditions such as such as ischemia/infarction, demyelination, encephalitis, ect (8, 13, 14). However, the authors assert that this characteristic is nonspecific and does not effectively differentiate germinomas from other basal ganglia tumors in children. These authors found that basal ganglia astrocytomas can also show similar atrophic changes (13). In comparison to the NGGCT, the current study found that basal ganglia germinomas were more likely to cause Wallerian degeneration, although there was no significant difference in internal capsule involvement between the two subtypes. This finding can be explained by histological studies, which have confirmed that germinomas high proliferative activity and low collagen deposition, leading to infiltrative growth (8).

During the progression of the disease, germinomas can present as irregular solid masses with cystic changes. Intratumoral tiny cysts may develop, although the overall tumor size remains unchanged. Perilesional edema and mass effects are disproportionate to tumor size. There is moderate to strong but heterogeneous enhancement after contrast agent administration. Intratumoral hemorrhage is common. Ipsilateral Wallerian degeneration is also a feature of germinomas in the basal ganglia (2, 3, 14). The solid component of the tumor typically has low apparent diffusion coefficient (ADC) values, reflecting the histological characteristics of germinomas, such as dense cellularity, a high nucleocytoplasmic ratio, and low water content (8). At this stage, it is crucial to consider basal ganglia glioma as a differential diagnosis. While germinomas are more frequently observed in adolescents and gliomas typically occur in older individuals, their clinical presentations can be notably similar. The lower prevalence of germinoma compared to glioma further complicates the differentiation between these two tumor types (10). Previous reports indicate that basal ganglia germinomas often present with minimal peritumoral edema that is disproportionate to the tumor size. In contrast, gliomas, particularly high-grade gliomas, are typically characterized by significant peritumoral edema (10).

Furthermore, advanced MRI images also aid in diagnosis. On tractography images, Wallerian degeneration is common, reflecting the infiltrative growth pattern of germinomas (8). This can be considered a reliable finding of basal ganglia germinomas. DSC-PWI images show lower rCBV and time-to-peak values (1, 17). This differentiates it from gliomas, which typically have a higher rCBV, reflecting neovascularization. MRS images demonstrate increased choline and decreased NAA, which helps distinguish neoplastic from non-neoplastic lesions. An increase in the lipid peak and taurine peak may be observed, although these features are not specific and are not always present (1, 17, 18).

In our patient, the initial MRI performed a year ago at another facility showed nonspecific abnormalities, which did not raise suspicion of a tumor. The subtle and nonspecific nature of the imaging findings, coupled with a lack of familiarity with early tumor indicators, likely contributed to the radiologists’ low index of suspicion. Consequently, the patient was not subjected to further evaluation and did not receive an earlier diagnosis or treatment. One year later, his brain MRI revealed a mass with more distinct features, as previously described, facilitating a more straightforward diagnosis. The patient subsequently underwent a biopsy and received appropriate treatment. However, despite the intervention, his symptoms only partially improved. Germinomas are known to respond well to chemotherapy and radiotherapy, typically resulting in a favorable prognosis and high survival rates. Early diagnosis and timely treatment often lead to complete or near-complete remission of the primary tumor, with long-term survival and, in some cases, complete neurological recovery (3, 6, 16). However, delayed diagnosis can result in tumor dissemination through local or cerebrospinal fluid (CSF) spread, similar to germinomas in the pineal or suprasellar regions. Thus, early imaging and histopathological diagnosis are essential for initiating timely treatment in patients with basal ganglia germinoma and minimizing the risk of neurological deficits (3, 6, 16).

In conclusion, the basal ganglia are very uncommon sites for germinomas. Accurate preoperative diagnosis helps avoid more invasive surgery. In addition, early identification of germinomas is essential to avoid the spread of the tumor and improve patient outcomes. The early diagnosis of basal ganglia germinoma is particularly challenging due to due to its rarity, the overlap of clinical symptoms with common neurological disorders, and the presence of subtle or atypical imaging features, leading to delays in diagnosis. Recognizing and being familiar with the characteristics of basal ganglia germinomas contributes to arousing suspicion when dealing with a lesion with the aforementioned findings, especially in male adolescents.

## Data Availability

The original contributions presented in the study are included in the article/supplementary material. Further inquiries can be directed to the corresponding author.
